# New challenges in psycho‐oncology: Using drug development methodology to improve survivorship and supportive care intervention trials

**DOI:** 10.1002/pon.5100

**Published:** 2019-05-20

**Authors:** Lesley Howells, Nicholas J. Hulbert‐Williams, Sarah P. Blagden

**Affiliations:** ^1^ Research Team Maggie's Centres London UK; ^2^ School of Psychology University of Chester Chester UK; ^3^ Department of Oncology University of Oxford Oxford UK

**Keywords:** cancer, clinical trial, phase 3, distress, oncology, ovarian cancer, OVPSYCH, psychosocial, quality of life, randomized controlled trial, survivorship

## 1 INTRODUCTION

Although a number of professional organisations recommend the routine screening of cancer patients for emotional distress, this is infrequently conducted as many centres lack suitable psychosocial interventions to offer those identified as being in distress.^1^, ^2^, ^3^, ^4^, ^5^ It is therefore essential to improve the evidence base around specific and effective interventions that can later be incorporated into routine cancer care. To provide sufficiently rigorous data supporting the benefit of any survivorship intervention, studies should be sized to allow adequate statistical powering and employ methodologies such as randomisation. Unfortunately, the majority of psychosocial/survivorship intervention studies do not adopt these methodologies due to challenges in funding and recruitment.^5^, ^6^, ^7^

**Figure 1:**
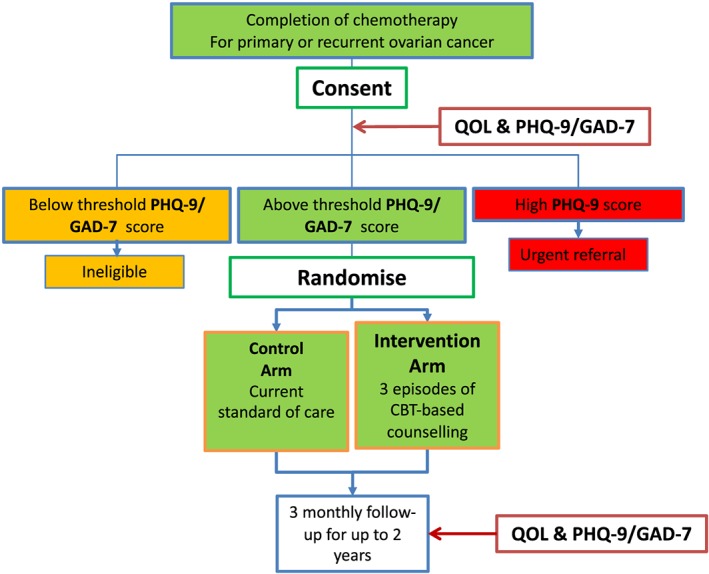
OVPSYCH trial design. OVPSYCH is a randomised controlled study to assess the impact of a psychosocial intervention following chemotherapy for advanced or recurrent OC. Potentially eligible patients were provided with study information at their final chemotherapy session (of their treatment course for newly diagnosed or relapsed OC). They were then invited to consent at their first follow‐up visit, approximately 4 to 6 weeks later and completed questionnaires to confirm their eligibility. Those scoring above a threshold on PHQ‐9 and/or GAD‐7 questionnaires were randomised 1:1 to the intervention or standard of care. Those in the intervention group were invited to receive three sessions of psychological support during the following 3 months. Those in the control arm received standard of care and were only referred for specialist supportive services (but not the OVPSYCH intervention) if they requested it or had symptoms of concern. Patients in both study arms then attended for routine outpatient follow‐up, over the subsequent 3 months for up to 2 years. At each of these appointments, patients were invited to complete QOL, PHQ‐9, and GAD‐7 questionnaires. This trial design was piloted in the OVPSYCH1 study.

Arguably, the best utilisation of randomised clinical studies occurs in clinical drug development whereby results from at least one Phase III drug development trial (P3DDT) are required for the licensing and approval of any new pharmaceutical agent.^8^ Important components of any P3DDT include defining the criteria (such as clinical characteristics and biomarkers) to select suitable patients, deciding the dose and duration of the investigational agent to be tested, developing a randomisation scheme and suitable control arm, designing endpoints, and incorporating safety reporting.^9^ These elements of a P3DDT are usually informed by preceding Phase I and II trials, appropriate theoretical modelling and pilot/feasibility studies.^10^ To explore whether P3DDT methodology could be translated to the design of a survivorship study, we incorporated it in the development of the OVPSYCH trial, to evaluate the impact of a psychosocial support intervention in patients with ovarian cancer (OC). The hypothesis of OVPSYCH was that post‐chemotherapy psychosocial support improves the psychological and overall well‐being of OC patients. We first conducted the pilot, OVPSYCH1, to which we recruited 32 patients. OVPSYCH1 was approved by Bradford Research Ethics Committee in 2011 (ref 11/YH/0117). Our findings from this pilot informed the design of OVPSYCH2, the first randomised study of post‐chemotherapy psychosocial support to be conducted in UK ovarian cancer patients. The overall design of the OVSYCH studies is summarised in Figure 1.

Below, we outline some of the key features of P3DDT methodology that were adapted for OVPSYCH.

### 1.1 Using biomarkers for patient selection

P3DDTs, where possible, use a biomarker to select or enrich for patients most likely to benefit from the study intervention. An example is Phase III lung cancer trials where only patients with tumour EGFR mutations were randomised for EGFR‐targeted therapy.^11^ In OVPSYCH, we decided to screen OC patients for psychosocial morbidity, randomising only those who scored over a defined threshold. Screening tools used previously have included a Distress Thermometer^12^ or Cancer Worry Scale,^13^ but as we could not predict whether depression or anxiety would predominate in our study patients, we chose to screen using both the Patient Health Questionnaire‐9 (PHQ‐9) depression and Generalized Anxiety Disorder (GAD‐7) questionnaires. These questionnaires have been demonstrated to be sensitive and reliable tools for cancer survivorship.^14^, ^15^ Patients also completed EORTC QLQ‐C30 and OV28 questionnaires so that any depression/anxiety scores could be correlated with general Quality of Life (QOL) measures.

The PHQ‐9 and GAD‐7 scores were developed by psychiatrists to screen and detect depression and anxiety respectively; scores on either scale of ≤4 indicate no symptoms, whereas scores 5 to 9 indicate mild and 10 to 14 moderate depression/anxiety. For the GAD‐7 scale, scores above 15 (of a 21‐score maximum) represent severe anxiety. For the PHQ‐9 scale, a score from 15 to 19 represents moderately severe depression, but scores ≥20 (of a 27‐score maximum) indicate severe depression. In addition, an affirmative answer to question 9 signals suicidal intent.^16^, ^17^ Based on a previous study where 60% OC patients reported moderate or severe psychological symptoms using EORTC QLQ‐C30 and Q28 scores,^18^ we initially aimed to recruit only patients with PHQ‐9 and/or a GAD‐7 scores ≥10. However, during the pilot, we discovered that when precise symptom‐specific questionnaires were implemented, depression/anxiety scores were lower than expected with only one third of patients with PHQ‐9 scores ≥10. We therefore lowered the cut‐off to include patients with mild, moderate, or moderately‐severe depression, corresponding to a PHQ‐9 score ≥4. In these patients, GAD‐7 scores were generally also elevated, but none had positive GAD‐7 and negative PHQ‐9 measures. We concluded that the PHQ‐9 was a better stratifier of emotional distress than GAD‐7.

Unlike in a P3DDT where patients with highest levels of a biomarker have greatest theoretical likelihood of response to the study intervention, in OVPSYCH, those with the highest “biomarker scores” such as PHQ‐9 scores ≥20 (denoting severe depression) or those that ticked question 9 of the PHQ‐9 scale were at highest risk of self‐harm. As these patients would be expected to enter a randomisation process that could result in them being assigned to “non‐intervention”, we mandated in the protocol that these patients were removed from the study and immediately referred for psychiatric or psychological support.

In identifying a patient stratification marker, in this case PHQ‐9, we found that symptom‐specific, quantitative questionnaires provided the best means of selecting patients. However, using these more discriminatory tools yielded a lower than expected number of eligible patients compared to less specific QOL scores. We concluded that eligibility range must be carefully defined and, ideally, previously validated in a similar patient population. Unlike P3DDTs, in survivorship studies, patients who have very high scores in symptom‐specific questionnaires may be unsafe for randomisation. In this situation, an appropriate treatment plan should be in place for those scoring over a safe limit.

### 1.2 Ensuring a valid control group

In P3DDTs, patients are usually randomised to either the intervention or control arm. Controls receive a placebo (in a blinded study) or an equivalent active/standard of care treatment. In the context of a survivorship study, designing a control arm can be challenging. As those in the intervention arm of OVPSYCH were requested to come into the hospital for additional counselling sessions, it would have been unethical to expect control patients to attend hospital for an equivalent number of visits but receive no counselling (placebo). But equally, there is insufficient evidence for the effectiveness of any specific type of psychosocial therapy to offer as an “equivalent active” control. We selected baseline service provision as the control against which the intervention arm could be compared. However, as there is no UK standard survivorship provision, this could vary between centres. As OVPSYCH1 was piloted in a single centre, we defined the activities in the control arm as “referral for survivorship support on demand or if expressing symptoms of concern.”

Once randomisation was in place, we observed another bias that has also been described in P3DDTs.^19^ Patients who agreed to enter OVPSYCH1 were generally more open to discussion about their psychological welfare and sought other means of support if they were randomised to the non‐intervention arm. We therefore introduced an “other treatments” form so trials staff could document any changes to medications or commencement of other supportive therapies (such as counselling or antidepressants) during the study period. Data from these forms were included in the statistical analysis at study closure.

In all randomised studies, control patients may be motivated to seek a similar intervention to those in the investigative study arm. This is easier for patients to achieve in a survivorship study than in a trial of a novel investigational agent. We recommend that survivorship studies are powered to allow some contamination and that patients are encouraged to disclose “other treatments” during their study follow‐up. In addition, trials may benefit from measuring access and treatment requirements from an equivalent, non‐trial group prior to the start of recruitment to provide a reliable comparator. This could, for example, be achieved by referencing an observational study documenting the uptake of supportive interventions in a similar patient population.

### 1.3 Providing safety measures

P3DDTs include harm‐related data reporting.^20^ Among other requirements, such as informing patients of possible risks of an intervention, study sites are required to submit timely Adverse Event (AE) Reports to the sponsors if study‐related side effects occur. Events such as admissions to hospital for worsening depression or death due to suicide are not inconceivable in cancer patients, and it is possible that the trial intervention (cognitive behavioural therapy [CBT]‐based counselling in OVPSYCH) is unsuitable for or may exacerbate the psychological concerns of patients.^21^ We therefore included an Adverse Event Reporting system within OVPSYCH so that any AEs would be noted and that suitable protective actions were taken to participants if AEs were caused by the study intervention. Along with a robust AE reporting system, a Trial Management Group (TMG) should be convened to periodically review adverse events and provide an additional layer of oversight to protect participants and investigators. If necessary, the TMG should have the power to terminate the trial on safety grounds.

Rather than being merely procedural, it is important to include an AE reporting system when designing a randomised survivorship trial. A TMG is also necessary to provide oversight and ensure intervention‐related AEs are followed with suitable protective actions to participants. Not only does this ensure the safety of study participants and the integrity of investigators but also provides important information that will be applicable to the later adoption of an intervention into clinical practice.

### 1.4 Defining study endpoints

Fundamental to clinical studies is their primary and secondary endpoints and the metrics used to assess them. The most commonly chosen primary endpoints for P3DDTs are progression‐free (PFS) and overall survival (OS); whereas patient experience is often chosen as a secondary endpoint and assessed using QOL questionnaires. In survivorship studies, the emphasis is reversed with primary endpoints being QOL or symptom‐specific measures. Although PFS and OS are valid secondary endpoints in survivorship studies, particularly as psychological well‐being has been shown to contribute to lifespan,^22^ long‐term follow‐up of patients to gather their survival data has cost implications. For this reason, and because we predicted that survival was unlikely to have been impacted by our intervention, we did not collect survival data in OVPSYCH and chose to set a change in PHQ‐9 as our primary endpoint.

As there are conflicting data from small longitudinal studies exploring the QOL trajectory in OC patients after chemotherapy, we predicted that psychosocial scores would deteriorate during chemotherapy treatment and remain poor in the following months.^23^, ^24^ To statistically power OVPSYCH, we predicted the PHQ‐9 score would be elevated immediately after chemotherapy in both arms but fall to a lower level in the intervention arm. We then considered the time required to capture our endpoint. Questionnaire‐based trials often show a drop‐off in completion rates over time. As follow‐up visits were three monthly for 2 years, we predicted poor questionnaire return and chose to measure data at the earliest (3 months) time point for our primary endpoint and defined significance as a ≥5 change (from baseline) in mean PHQ‐9 score in the intervention compared to the control arm. Secondary analyses include comparisons of PHQ‐9 and between‐group comparisons using the other scores (GAD‐7, EORTC QLQ‐OV28, and QLQ‐C30) at later time points. However, we recognised that lack of questionnaire return may result in these secondary endpoints being unmet.

We conclude that, unlike in P3DDTs, QOL studies need to assume poor questionnaire compliance and focus their primary endpoints around early time points. If funding is limited, endpoint measures should be directly appropriate to the intervention aims rather than focusing on biological progression or survival. However, if longer‐term follow‐up data are essential, investigators could consider direct methods of follow‐up with participants, such as by telephone, to circumnavigate institutional limitations such as difficulties in collecting questionnaires from participants.

### 1.5 Defining and standardising the intervention

An important component in a P3DDT is ensuring the intervention is standardised, in the case of trial of a therapeutic agent, in dose and schedule. We initially intended that the intervention, a course of CBT‐based psychosocial support sessions, was provided by the hospital‐based survivorship service. However, this service is non‐standardised across the United Kingdom and is provided by counsellors, psychologists, or psychiatrists and with different approaches (such as antidepressants, CBT, mindfulness, etc), in individual sessions or in groups.

In the pilot study, we opted for a standard “dose” of three 90‐minute sessions of CBT‐based counselling, to be provided in the 3 months between the first and second follow‐up visit. So as not to interfere with daily outpatient activities, patients were invited to early‐evening appointments with the departmental counsellor in the chemotherapy unit. Although the session content was not strictly predefined, it was designed to cover a number of specific topics such as how to manage anxiety, stress, depression, and anger and included broader well‐being issues such as relationships, diet, and ways to manage day‐to‐day living. However, amongst those randomised to receive the intervention, attendance was poor. Qualitative feedback indicated that patients were reluctant to return to the place where they had received chemotherapy, and the early‐evening timing of the sessions was also unpopular, particularly for those experiencing post‐chemotherapy fatigue.

We concluded that the venue for a psychosocial intervention is vital, the intervention should be conducted at a time that suits the patients and at a location that does not have negative associations. As there is considerable variability in experience and training of those providing survivorship support in cancer hospitals, we realised that the standardisation of any psychosocial intervention would be challenging, both between participants within one centre and across multiple centres. We therefore sought a tertiary, independent care centres (Maggie's Cancer Centres) to provide a standardised psychosocial intervention for our main OVPSYCH study. In our pilot, treatment fidelity was poor. We therefore propose that for psychosocial intervention studies, a record of attendance should be maintained by those providing the intervention and that this is included in any data collection on study completion. Moreover, poor compliance should be anticipated to prevent statistical underpowering.

## 2 CONCLUSIONS

In order to improve the quality of survivorship support available to cancer patients, studies must provide a robust evidence‐base for interventions that can be adopted into clinical practice.^6^ We have shown here that applying methodology developed for P3DDTs is useful in terms of randomisation strategies, adverse event reporting, patient stratification, and the selection and standardisation of study arms. However, there are several important limitations. P3DDTs include long‐term survival data as primary endpoints, but in survivorship trials where funding is invariably limited, short‐term primary endpoints are preferred to accommodate diminishing questionnaire return and to limit the expense of patient follow‐up. In addition, patient co‐morbidities and even death from underlying disease will contribute to poor data return. Thus, survivorship studies, even when randomised, may fail to explore and address the impact of an intervention on the longer‐term psychological morbidity and survival of study patients. Institutional apathy is a recurrent limitation to conducting survivorship research, and the onus to participate and complete information sheets can fall on the participants themselves. It is therefore vital that any proposed intervention is fully acceptable to participants to ensure their motivation and compliance.

Uncertainties around the natural psychosocial trajectory after cancer treatment along with limited standardised survivorship provision in cancer centres create an unreliable baseline against which novel interventions must be compared. With the emergence of P3DDTs evaluating maintenance therapies in the OC setting and publishing their QOL data alongside the main trial results, our understanding of the long‐term QOL trajectory in OC patients is improving. However, these patients are preselected by performance status and other (eg, biochemical) parameters and are not necessarily representative of those seen in standard of care settings. These data should be interpreted with caution when designing psychosocial intervention studies.

As cancer survival improves and greater numbers of patients require evidence‐based psychosocial support, the need for good quality survivorship research will intensify. Survivorship studies are challenging to conduct for a number of well‐documented reasons, including cost limitations and lack of institutional interest or support.^12^, ^25^ This creates a chicken‐and‐egg situation whereby there is an insufficient evidence‐base from which to implement standardised survivorship programmes in cancer centres. In the context of cancer distress, this can lead to long‐term psychological morbidity in patients, resulting in failure to return to work and chronic demands on health care resources.^26^ In an attempt to break this cycle, we applied P3DDT methodology to the design of a survivorship intervention study, OVPSYCH2. Here, we described the pilot study OVPSYCH1, conducted to determine the limitations of this approach. These findings were subsequently incorporated into the design of the main OVPSYCH2 trial, a multicentred randomised study of a psychosocial supportive intervention for OC patients following chemotherapy.

## CONFLICTS OF INTERESTS

The authors have no conflicts of interest to report.
